# Reliability of spatiotemporal and kinetic gait parameters determined by a new instrumented treadmill system

**DOI:** 10.1186/1471-2474-14-249

**Published:** 2013-08-21

**Authors:** Lloyd F Reed, Stephen R Urry, Scott C Wearing

**Affiliations:** 1 School of Clinical Sciences, Queensland University of Technology, Kelvin Grove, 4059 Queensland, Australia; 2 Institute of Health and Biomedical Innovation, Queensland University of Technology, 60 Musk Avenue, Kelvin Grove, 4059 Queensland, Australia; 3 Faculty of Health Sciences and Medicine, Bond University, Gold Coast, 4229 Queensland, Australia; 4 Centre of Excellence for Applied Sport Science Research, Queensland Academy of Sport, PO Box 956, Sunnybank, 4109 Queensland, Australia

**Keywords:** Gait analysis, Walking, Measurement, Repeatability, Precision, Reproducibility

## Abstract

**Background:**

Despite the emerging use of treadmills integrated with pressure platforms as outcome tools in both clinical and research settings, published evidence regarding the measurement properties of these new systems is limited. This study evaluated the within– and between–day repeatability of spatial, temporal and vertical ground reaction force parameters measured by a treadmill system instrumented with a capacitance–based pressure platform.

**Methods:**

Thirty three healthy adults (mean age, 21.5 ± 2.8 years; height, 168.4 ± 9.9 cm; and mass, 67.8 ± 18.6 kg), walked barefoot on a treadmill system (FDM–THM–S, Zebris Medical GmbH) on three separate occasions. For each testing session, participants set their preferred pace but were blinded to treadmill speed. Spatial (foot rotation, step width, stride and step length), temporal (stride and step times, duration of stance, swing and single and double support) and peak vertical ground reaction force variables were collected over a 30–second capture period, equating to an average of 52 ± 5 steps of steady–state walking. Testing was repeated one week following the initial trial and again, for a third time, 20 minutes later. Repeated measures ANOVAs within a generalized linear modelling framework were used to assess between–session differences in gait parameters. Agreement between gait parameters measured within the same day (session 2 and 3) and between days (session 1 and 2; 1 and 3) were evaluated using the 95% repeatability coefficient.

**Results:**

There were statistically significant differences in the majority (14/16) of temporal, spatial and kinetic gait parameters over the three test sessions (P < .01). The minimum change that could be detected with 95% confidence ranged between 3% and 17% for temporal parameters, 14% and 33% for spatial parameters, and 4% and 20% for kinetic parameters between days. Within–day repeatability was similar to that observed between days. Temporal and kinetic gait parameters were typically more consistent than spatial parameters. The 95% repeatability coefficient for vertical force peaks ranged between ± 53 and ± 63 N.

**Conclusions:**

The limits of agreement in spatial parameters and ground reaction forces for the treadmill system encompass previously reported changes with neuromuscular pathology and footwear interventions. These findings provide clinicians and researchers with an indication of the repeatability and sensitivity of the Zebris treadmill system to detect changes in common spatiotemporal gait parameters and vertical ground reaction forces.

## Background

Treadmill walking is emerging as a viable intervention for treating gait impairments following neurological disorders such as stroke [1], spinal cord injury [2], and Parkinson’s disease [3]. Over the last 20 years, instrumented treadmills that incorporate one or more high–fidelity force plates have emerged as a valuable measurement tool in clinical gait studies and applied research settings [4-6]. Instrumented treadmills provide basic spatiotemporal gait parameters and ground reaction forces in near real–time and have been reported to have ‘high’ levels of reliability, with coefficients of variation (CVs) of < 10% typically reported between– and within–days for temporal gait parameters and ground reaction forces (GRF) collected with these systems [7,8].

Recently, however, a relatively new instrumented treadmill that incorporates an array of pressure transducers, rather than a force plate, has become commercially available. To date, this new system has been used to investigate fundamental control mechanisms in gait [9,10], disturbances associated with neurological disorders, including Parkinson’s disease and cerebellar ataxia [11-13], and as an outcome measure to monitor the progression of ergonomic training programs [14], and the effectiveness of various clinical [15], and neuro–rehabilitation trials [16].

Despite the increasing use of these instrumented treadmills in clinical and research settings, however, there is limited published data regarding their measurement properties. This is surprising, given the performance characteristics and spatial resolution of similar capacitance–based pressure platforms are known to differ to those of force platforms [17]. In one of the few studies performed to date, Faude et al. [14] reported that CVs between–days were typically < 7% for most parameters, except for measures of temporal (25–30%) and spatial (32–36%) variability in healthy seniors (n = 20; mean age, 64.3 ± 3.2 years) when walking at a constant imposed walking speed (1.39 m.s^-1^). However, the study did not evaluate the repeatability of GRF–based parameters, which are also routinely derived by these systems.

The aim of the current investigation, therefore, was to evaluate the within– and between–day repeatability of spatiotemporal gait parameters and vertical ground reaction forces measured in a group of healthy young adults while walking at self–selected speeds on a capacitance–based treadmill system. We specifically evaluated the repeatability of basic gait parameters in young adults as recent intervention studies have used the same treadmill system as outcome measures in this cohort [15,18].

## Methods

Thirty three (9 male and 24 female) healthy adults were recruited from University faculty to participate in the study. The mean (± SD) age, height, mass and body mass index of participants was 21.5 ± 2.8 years, 168.4 ± 9.9 cm, 67.8 ± 18.6 kg, and 23.9 ± 6.1 kg.m^-2^, respectively. No participant reported a history of medical or balance disorders or musculoskeletal conditions likely to affect their ability to walk on a treadmill. All participants gave written informed consent prior to participation in the research. The study received approval from the university human research ethics committee and was undertaken according to the principles outlined in the Declaration of Helsinki.

Participants reported to the gait laboratory wearing lightweight, comfortable clothing and having abstained from vigorous physical activity on the day of testing. Following anthropometric assessment, participants were instructed to walk barefoot at their ‘preferred’ walking speed over a 10–m walkway which incorporated an instrumented pressure mat (GAITRite system, CIR Systems Inc., 60 Garlor Drive Havertown, PA 19083). To negate the effects of gait initiation and termination, gait speed was determined over the central 4.8 m of the walkway and averaged over ten gait trials.

**Figure 1:**
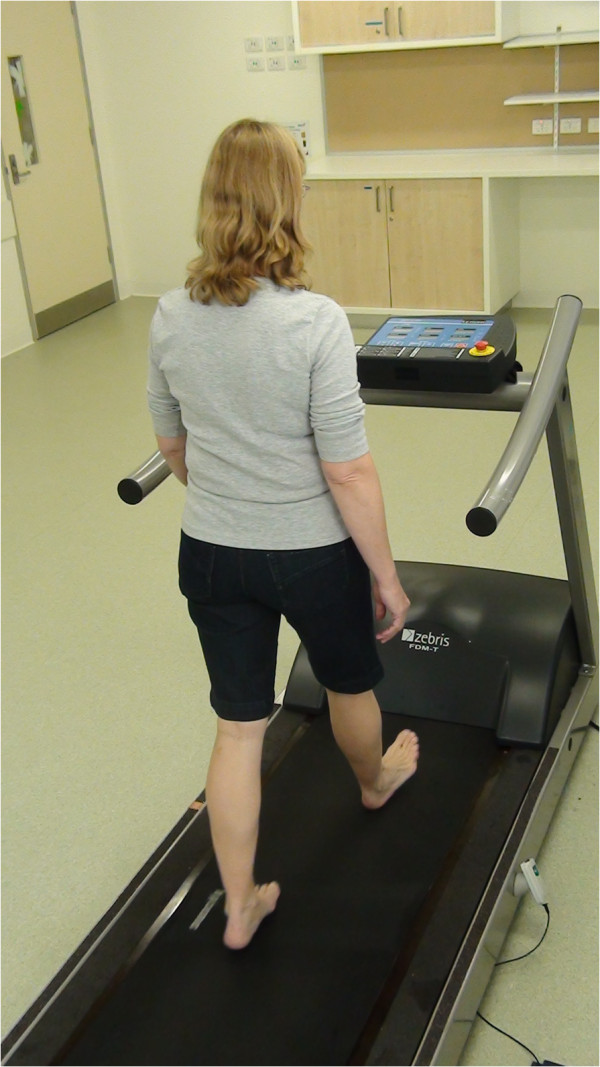
**Instrumented treadmill system.** Spatiotemporal gait parameters and ground reaction forces were estimated using an instrumented treadmill system that incorporated a capacitance–based pressure array consisting of 7,168 transducers with a spatial resolution of 0.85 cm.

Participants were then requested to walk barefoot on the Zebris FDM–THM–S treadmill system (Zebris Medical GmbH, Max–Eyth–Weg 43, D–88316, Isny, Germany) on three separate occasions. The system was comprised of a capacitance–based pressure platform housed within a treadmill. The pressure platform had a sensing area of 108.4 × 47.4 cm and incorporated 7,168 sensors, each approximately 0.85 × 0.85 cm. The treadmill had a contact surface of 150 × 50 cm and its belt speed could be adjusted between 0.2 and 22 km.h^-1^, at intervals of 0.1 km.h^-1^ (Figure 1). The grade of the contact surface of the treadmill was maintained in a horizontal position (0%) throughout testing. As outlined by Van de Putte et al. [19], participants were afforded a treadmill acclimatization session, in which they were briefed regarding the safety procedures and provided with a 10 minute familiarization period of walking at self–selected speed. Following the acclimatization session, participants advised a member of the research team to steadily increase the treadmill speed in increments of 0.1 km.hr^-1^ until they first reported they exceeded their preferred “comfortable” walking pace. Speed was then decremented by 0.1 km/h until the participant confirmed that their preferred “comfortable” walking pace was re–established. After 3 minutes of walking, gait data were captured over a 30 second period, equating to an average of 52 ± 5 steps. A sampling rate of 120 Hz was used to acquire all data. Testing was repeated one week following the initial trial and was repeated again, for a third time, 20 minutes later; allowing an evaluation of between–day and within–day variability in gait characteristics, respectively. Given that variability in gait parameters is typically lowest at self–selected gait speeds [20], participants were instructed to set a comfortable walking pace for each testing session, but were blinded to the selected treadmill speed.

**Figure 2:**
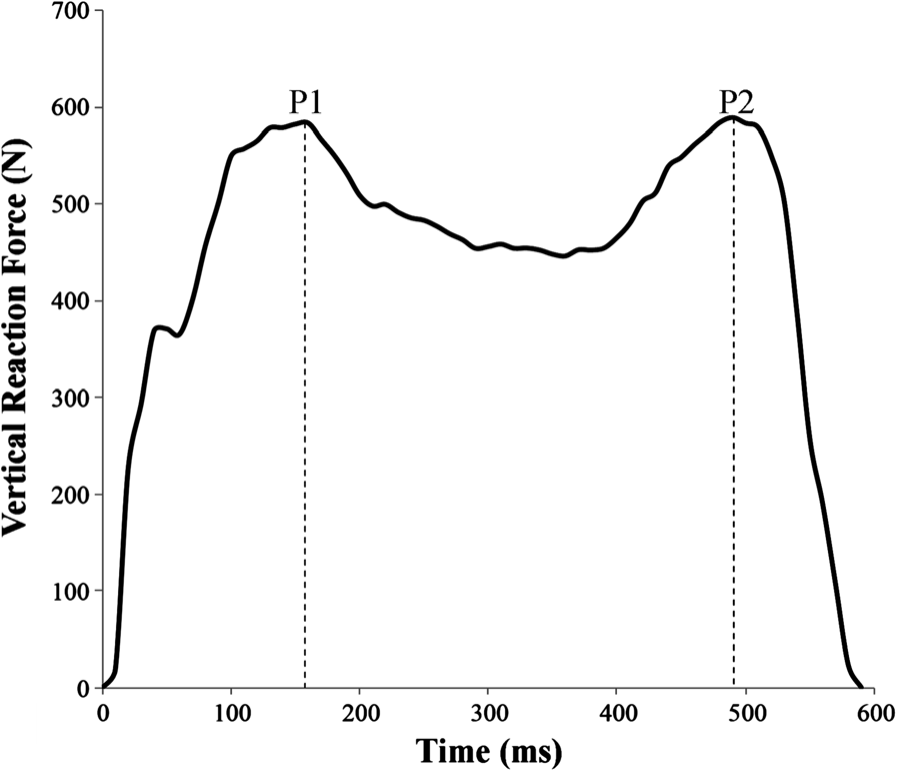
**Illustration of a typical vertical ground reaction force–time trace obtained with the instrumented treadmill.** The magnitude and timing of the vertical ground reaction force braking peak (P1), and final propulsive peak (P2) were calculated for comparison across the three test sessions. Time to peak force was expressed as a percentage of the stance phase duration.

Proprietary software was subsequently used to calculate mean spatiotemporal gait parameters (Table 1), including cadence, step and stride length, step width, foot rotation, stance and stride times and swing phase and single and double support durations. With the exception of stride and stance times, temporal data were expressed as a percentage of the gait cycle. Vertical ground reaction force data were exported in ASCII format and custom computer code (Matlab R2012a, MathWorks, Natick, MA) was subsequently used to identify the magnitude and timing of conventional vertical ground reaction force peaks for each step (Figure 2) [21]. The relative time to peak force was expressed as a percentage of the stance phase duration and mean peak force values were calculated.

**Table 1:** Average (SD) temporal and spatial gait parameters (n = 33)

	**Session 1**	**Session 2**	**Session 3**	**P**^**†**^
**Global**				
Velocity, m/s	1.10	1.13	1.24 *	< 0.001
	(0.17)	(0.19)	(0.19)	
Cadence, steps/min	110.3	111.3	115.7 *	< 0.001
	(9.8)	(9.6)	(8.8)	
**Spatial**				
Stride length, cm	119.2	121.1	128.4 *	< 0.001
	(14.1)	(15.6)	(14.7)	
Step width, cm	8.7	9.0	8.6	.115
	(3.0)	(2.4)	(2.4)	
Step length, left, cm	59.6	60.7	64.3 *	< 0.001
	(7.4)	(8.2)	(7.5)	
Step length, right, cm	59.6	60.4	64.1 *	
	(6.8)	(7.4)	(7.3)	
Foot rotation, left, º	8	7	7	.371
	(5)	(5)	(5)	
Foot rotation, right, º	9	9	9	
	(5)	(6)	(5)	
**Temporal**				
Stride time, ms	1097	1087	1043 *	< 0.001
	(102)	(92)	(79)	
Step time, left, ms	548	544	522 *	< 0.001
	(51)	(48)	(41)	
Step time, right, ms	549	542	521 *	
	(51)	(45)	(38)	
Stance phase, left, %	63	63	62 *	< 0.001
	(2)	(2)	(1)	
Stance phase, right, %	63	63	62 *	
	(2)	(2)	(2)	
Swing phase, left, %	37	37	38 *	< 0.001
	(2)	(2)	(1)	
Swing phase, right, %	37	37	38 *	
	(2)	(2)	(2)	
Single support, left, %	37	37	38 *	< 0.001
	(2)	(2)	(2)	
Single support, right, %	37	37	38 *	
	(2)	(2)	(1)	
Double support, left, %	26	25	24 *	< 0.001
	(3)	(3)	(3)	
Double support, right, %	26	25	24 *	
	(3)	(3)	(3)	

* significantly different from all other sessions (P < .01), ^**†**^ P value for main effect of test session.

The Statistical Package for the Social Sciences (SPSS Inc, Chicago, IL, USA) was used for all statistical procedures. All data were evaluated for normality using the Kolmogorov–Smirnov test. Differences between over–ground and treadmill walking speeds and between–session differences in treadmill walking speed, cadence, step width, stride length and stride time were assessed using repeated measures ANOVA within a generalized linear modelling framework. For all other parameters, two–way repeated measure ANOVA models with simple contrasts were employed to investigate main effects for walking session. In each case, session (1–3) and limb (left, right) were treated as within–subject factors. Underlying assumptions regarding the uniformity of the variance–covariance matrix were assessed using Mauchly’s test of sphericity. Given the number of statistical tests, an alpha level of .01 was used as a more conservative approach for tests of significance. Absolute variability between gait parameters measured within the same day (session 2 and 3) and between days (session 1 and 2; session 1 and 3) was estimated using the *Standard Error of Measurement* (SEM), where SEM = SD_d_/√2 and SD_d_ refers to the standard deviation of difference scores for individuals on the two occasions [22]. Agreement between gait parameters measured within– and between days were evaluated using the bias and *Repeatability Coefficient* (RC_95%_), as outlined by Bland and Altman [23]. The RC_95%_ represents the upper and lower limits between which two repeated measures will fall for 95% of participants and is given by the equation; RC_95%_ = 1.96 × SD_d_, where 1.96 reflects the zx–score associated with the desired level of confidence [23]. As calculated, the RC_95%_ is mathematically identical to the *Minimum Detectable Change* (MDC_95%_), which is frequently used within the rehabilitation literature to represent the minimum change in score (at an individual level) that likely reflects true change (with 95% confidence), rather than measurement error alone [22,24].

## Results

Average gait speed during over–ground walking (1.35 ± 0.14 m.s^-1^) was significantly faster than during treadmill walking (F = 15.0, P < .001). Mean spatial and temporal parameters for treadmill walking during the three gait sessions are presented in Table 1. With the exception of foot rotation angle and step width, there were statistically significant main effects for test session for all temporal and spatial gait parameters. In session 3, participants walked, on average, 0.13 m.s^-1^ faster, increased their cadence by 5 steps.min^-1^, and adopted stride lengths and step times that were around 6% longer and 5% shorter, respectively, than in sessions 1 and 2. Session three was also accompanied by a concomitant decrease in the duration of double support. There were no statistically significant differences, however, between the first and second gait trials for any spatiotemporal parameter.

Table 2 illustrates the topic maxima in the vertical GRF for the three test sessions. There was a significant main effect for the magnitude and timing of the vertical GRF braking peak and the final propulsive peak across the sessions. While the vertical propulsive peak was significantly lower in the first session compared to subsequent sessions, the braking peak was significantly higher in the final session when compared to the previous walking trials.

**Table 2:** Average (SD) kinetic gait parameters (n = 33)

	**Session 1**	**Session 2**	**Session 3**	**P**^**†**^
First force peak, left, N	715	718	728 *	.004
	(185)	(182)	(181)	
First force peak, right, N	724	726	742 *	
	(187)	(183)	(184)	
Second force peak, left, N	732 *	747	745	.001
	(169)	(168)	(167)	
Second force peak, right, N	739 *	756	754	
	(169)	(169)	(167)	
Time first force peak, left, %	17	16	15 *	< 0.001
	(2)	(2)	(2)	
Time first force peak, right, %	17	16	15 *	
	(2)	(2)	(2)	
Time second force peak, left, %	46*	46	46	.005
	(2)	(1)	(2)	
Time second force peak, right, %	46 *	46	46	
	(1)	(1)	(2)	

* significantly different from all other sessions (P < .01), ^**†**^ P value for main effect of test session.

**Figure 3:**
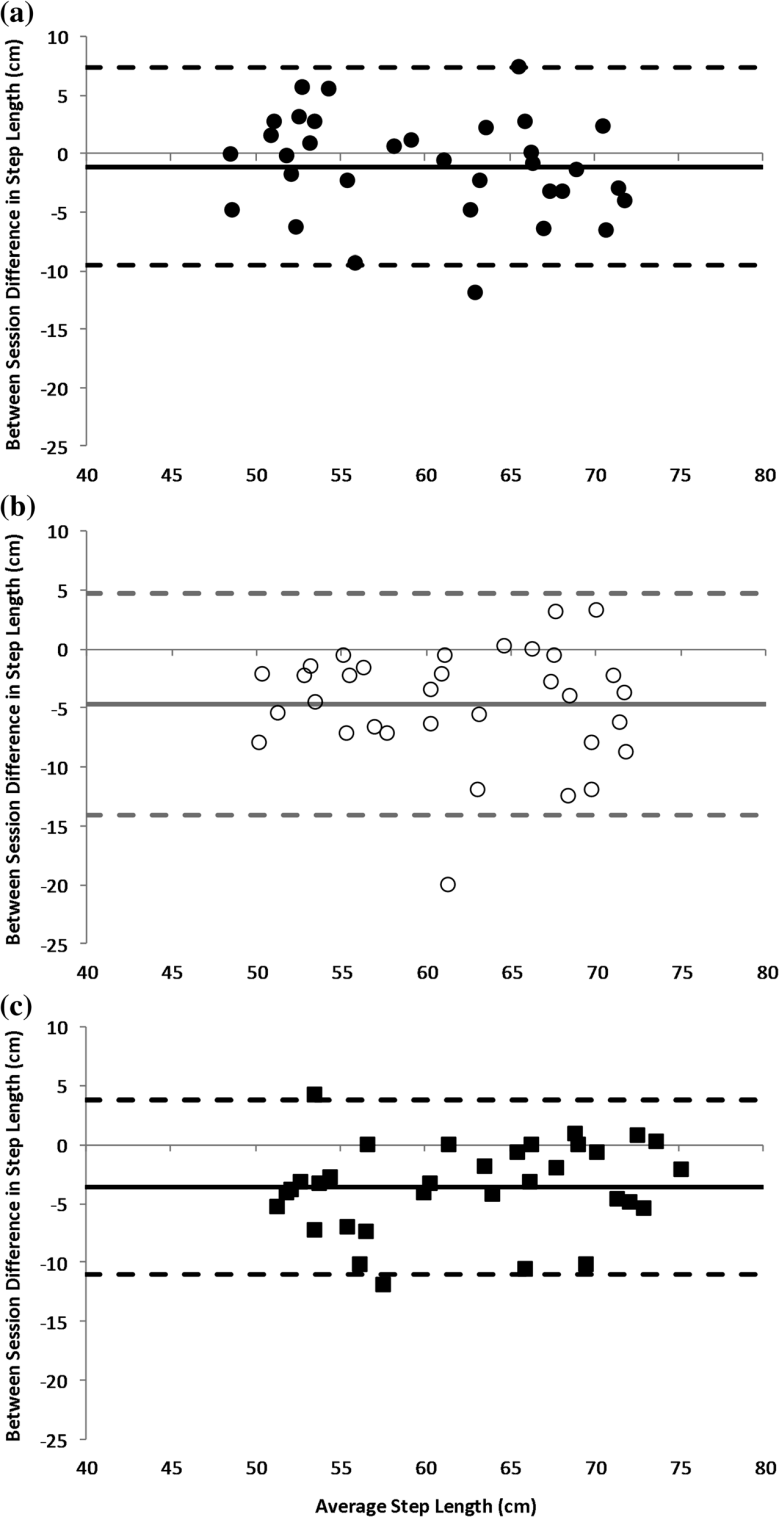
**Bland and Altman plot for step length of the left leg.** Bias (solid line) and RC_95%_ (dashed lines) for step length between sessions 2 and 3 (within–day) **(a)** and between sessions 1 and 2 (between–day) **(b)** and between sessions 1 and 3 (between–day) **(c)**. Note that the RC_95%_ is mathematically identical to the *Minimum Detectable Change*, which represents the minimum change in score (at an individual level) that reflects true change (with 95% confidence), rather than measurement error alone. Negative values reflect an increase in step length.

When expressed as a percentage of the mean, the SEM was less than 10% for the majority of gait variables, including step length (Figure 3), for both within– and between–day comparisons (Table 3). The exceptions were step width (SEM 10% of between–day mean), left foot rotation angle (SEM 14% of between– and within–day mean) and right foot rotation angle (SEM 11% of between– and within–day mean). Foot rotation angle and self–selected walking speed had the greatest variability of all gait variables (Table 3). Temporal gait parameters were typically more consistent than spatial measures and variability for all parameters was generally smaller within– rather than between–days. While the SEM of vertical ground reaction force peaks were less than 5% of the mean values for within– and between–day comparisons, the 95% limits of agreement ranged between ± 53 and ± 63 N (Table 4).

**Table 3:** Agreement among temporal and spatial gait parameters (n = 33)

	**Within day (S2 – S3)**		**Between day (S1 – S2)**		**Between day (S1 – S3)**	
	**SEM**	**Bias**	**SEM**	**Bias**	**SEM**	**Bias**
	**(SEM**_**%**_**)**	**(± RC**_**95%**_**)**	**(SEM**_**%**_**)**	**(± RC**_**95%**_**)**	**(SEM**_**%**_**)**	**(± RC**_**95%**_**)**
**Global**						
Velocity, m/s	0.07	−0.11	0.08	−0.03	0.10	−0.14
	(6)	(0.21)	(7)	(0.23)	(9)	(0.26)
Cadence, steps/min	3.3	−4.4	3.4	−0.9	4.1	−5.4
	(3)	(9.0)	(3)	(9.5)	(4)	(11.2)
**Spatial**						
Stride length, cm	5.1	−7.3	6.0	−1.9	6.6	−9.2
	(4)	(14.2)	(5)	(16.7)	(6)	(18.4)
Step width, cm	0.5	0.4	0.9	−0.3	0.9	0.1
	(6)	(1.4)	(10)	(2.5)	(10)	(2.4)
Step length, left, cm	2.7	−3.6	3.0	−1.1	3.4	−4.7
	(4)	(7.4)	(5)	(8.4)	(6)	(9.4)
Step length, right, cm	2.6	−3.7	3.0	−0.8	3.3	−4.5
	(4)	(7.1)	(5)	(8.4)	(6)	(9.1)
Foot rotation, left, º	1	0	1	0	1	0
	(14)	(3)	(14)	(2)	(14)	(2)
Foot rotation, right, º	1	0	1	0	1	0
	(11)	(3)	(11)	(2)	(11)	(3)
**Temporal**						
Stride time, ms	33	43	37	10	46	53
	(3)	(91)	(3)	(103)	(4)	(128)
Step time, left, ms	17	22	19	4	24	26
	(3)	(47)	(3)	(52)	(4)	(67)
Step time, right, ms	16	21	19	7	22	27
	(3)	(45)	(3)	(51)	(5)	(62)
Stance phase, left, %	1	1	1	0	1	1
	(2)	(2)	(2)	(2)	(2)	(2)
Stance phase, right, %	1	1	1	0	1	1
	(2)	(2)	(2)	(2)	(2)	(2)
Swing phase, left, %	1	−1	1	0	1	−1
	(3)	(2)	(3)	(2)	(3)	(2)
Swing phase, right, %	1	−1	1	0	1	−1
	(3)	(2)	(3)	(2)	(3)	(2)
Single support, left, %	1	−1	1	0	1	−1
	(3)	(2)	(3)	(2)	(3)	(2)
Single support, right, %	1	−1	1	0	1	−1
	(3)	(2)	(3)	(2)	(3)	(2)
Double support, left, %	1	2	1	1	1	2
	(4)	(3)	(4)	(4)	(4)	(4)
Double support, right, %	1	2	1	1	1	2
	(4)	(3)	(4)	(4)	(4)	(4)

*SEM* Standard error of measurement, *SEM*_*%*_ Standard error of measurement expressed as percentage of mean, *RC*_*95%*_ 95% repeatability coefficient.

**Table 4:** Agreement between kinetic gait parameters (n = 33)

	**Within day (S2 – S3)**		**Between day (S1 – S2)**		**Between day (S1 – S3)**	
	**SEM**	**Bias**	**SEM**	**Bias**	**SEM**	**Bias**
	**(SEM**_**%**_**)**	**(± RC**_**95%**_**)**	**(SEM**_**%**_**)**	**(± RC**_**95%**_**)**	**(SEM**_**%**_**)**	**(± RC**_**95%**_**)**
First force peak, left, N	17	−11	19	−3	20	−13
	(2)	(47)	(3)	(54)	(3)	(55)
First force peak, right, N	20	−15	24	−3	22	−18
	(3)	(55)	(3)	(66)	(3)	(62)
Second force peak, left, N	16	2	18	−15	19	−13
	(2)	(45)	(2)	(49)	(3)	(53)
Second force peak, right, N	16	2	27	−17	21	−14
	(2)	(43)	(4)	(52)	(3)	(59)
Time first force peak, left, %	1	1	1	0	1	2
	(7)	(3)	(6)	(3)	(7)	(3)
Time first force peak, right, %	1	1	1	0	1	2
	(7)	(2)	(6)	(3)	(7)	(3)
Time second force peak, left, %	1	1	1	0	1	1
	(2)	(2)	(2)	(1)	(2)	(2)
Time second force peak, right, %	1	0	1	0	1	0
	(2)	(2)	(2)	(2)	(2)	(2)

*SEM* Standard error of measurement, *SEM*_*%*_ Standard error of measurement expressed as percentage of mean, *RC*_*95%*_ 95% repeatability coefficient.

## Discussion

This study evaluated within– and between–day repeatability of spatiotemporal and kinetic gait parameters measured by an instrumented treadmill system that incorporated an array of pressure transducers, in a group of healthy young adults while walking at self–selected speeds. The SEM, when expressed as a percentage of the mean, was typically less than 10% for all gait parameters, except foot rotation angle and step width. Ten of the 16 test parameters possessed an SEM of less than 6% of the respective mean. Temporal parameters were generally the most consistent gait parameters both within– and between days, followed by ground reaction forces and then spatial parameters. Based on the findings of the current study, the minimum change that can be detected with 95% confidence for repeated measurements made on the same day varied between 3% and 13% for temporal parameters, 4% and 20% for kinetic parameters, and 11% and 43% for spatial parameters. Between–day repeatability of gait parameters was similar to within–day, with the minimum detectable change (95%) ranging between 3% and 17% for temporal parameters, 4% and 20% for kinetic parameters, and 14% and 33% for spatial parameters. Interestingly, similar levels of variability have been reported for temporal and spatial gait parameters during both over–ground (temporal, 1–8%; spatial, 6–31%) [25,26] and treadmill walking (temporal, 4–5%; spatial, 8–46%) in healthy young adults [27].

In a recent study, Faude et al. [28] reported reliability coefficients (CV < 7%) for spatiotemporal gait parameters in healthy seniors while walking on an instrumented treadmill at speeds matched to that determined during over–ground walking (1.39 m.s^-1^). However, they reported lower variability for repeated measures between days compared to within days. The present study differs from that of Faude et al. [28] in at least three important ways. First, walking speed was not matched to a predetermined over–ground walking speed and across all sessions in our study. Rather, participants were free to select a ‘comfortable’ walking speed during each session and were blinded to their selected speed. Hence, we were able to determine the repeatability of walking speed on the treadmill system. Interestingly, all participants in this study adopted a slower speed (13–23%) during treadmill walking compared to over–ground locomotion. It is well documented that speed and variability in basic gait parameters display a quadratic relationship in young adults walking at fixed treadmill speeds, where gait variability increases at speeds slower or faster than preferred [10-12]. Thus, by allowing participants to self select their speed, we anticipated that variability in gait parameters would be minimised. However, walking speed had the third highest variability of all gait parameters in the current study, both within and between days (SEM, 6–9%).

Second, Faude et al. [28] investigated reliability of basic gait parameters in a group of senior citizens, while we evaluated repeatability of basic gait parameters in healthy young adults. Although the variability of basic gait parameters has been reported to be higher in older adults, independent of walking speed [29], it is noteworthy that Faude et al. [28] reported lower variability estimates for all gait parameters in their study. Thus, it could be argued that constraining speed to a constant value for all test sessions may further improve reliability estimates. However, it should be noted that the variability in self–selected treadmill speed in this study (SEM, 0.07–0.1 m.s^-1^) is comparable to that noted during over–ground walking (SEM, 0.08 m.s^-1^) in healthy adults [25], and is also similar to the limits (± 0.2 m.s^-1^) imposed by some studies when investigating the effect of over–ground walking speed on gait parameters [30].

A third important difference between studies is that Faude et al. [28] afforded participants with a one minute acclimatization period prior to testing, while participants in the present study were afforded a 10 minute familiarization period. Previous research has recommended that a practice period of 10 minutes is required prior to testing to minimize potential learning effects associated with treadmill walking [19]. As demonstrated in Tables 1 &2, however, we observed that participants typically adopted a faster walking speed, higher step rate (cadence) and longer step length with increased exposure to treadmill walking. Moreover, the changes in spatiotemporal parameters were accompanied by an increase in vertical braking and propulsive forces over the three test sessions, suggesting that participants adopted a less tentative gait pattern with greater exposure to treadmill walking. Similar increases in step length have also been observed with habituation to constant speed treadmill walking [31]. It is also interesting to note that, despite imposing an identical walking speed across all test sessions, Faude et al. [28] observed consistently lower CVs in all gait parameters with increasing exposure to treadmill walking (ie across days 1, 2 & 3).

Although the reliability of spatiotemporal measures determined by the Zebris system has been described in healthy seniors [28], this is the first time, to our knowledge, that the repeatability of GRF–based indices has been assessed with this treadmill system. Based on the findings of this study, only differences in peak ground reaction forces in the order of ±63 N can be reliably detected with 95% confidence (Table 4). This limit is comparable to that recorded by an instrumented treadmill system incorporating a force plate (≈ 6%) in healthy young adults during walking [32]. It is noteworthy however, that recent research in which the same instrumented treadmill was used to evaluate the influence of footwear on gait, noted significant differences of the order of 20 to 70 N in the first vertical force peak between shod and unshod conditions [15]. While the research concluded that standard running shoes significantly increased impact force peaks, these differences fall within the 95% limits of agreement for braking and propulsive force peaks assessed in the present study; suggesting the effect may also reflect measurement error. Similarly Mak [33], using the same treadmill system, concluded that Parkinson’s disease primarily reflected a disturbance of step length regulation (rather than step variability) on the basis that step length was selectively reduced by 2 cm in Parkinson’s disease during a dual–task experiment. Others have reported comparable changes in step length with visual cuing interventions in this cohort [11]. These differences, however, fall clearly within minimum detectable change in step length found in the present study and highlight the need for the continued evaluation of the measurement characteristics of the instrumented treadmill systems in a variety of populations and over a wide range of gait speeds.

This study has a number of limitations which need to be considered when interpreting the results. Firstly, we evaluated the repeatability of common spatiotemporal and kinematic parameters in healthy young adults at a self–selected ‘comfortable’ walking speed. As such the findings may not be applicable to children, older cohorts, or individuals with gait abnormalities in which spatiotemporal parameters may vary markedly and faster or slower gait speeds are common [25,34]. Second, we allowed participants to self select their preferred walking speed during each session rather than impose a constant predetermined speed. While this may be viewed as a limitation, it allowed us to determine the repeatability of self–selected walking speeds on the treadmill system. Third, treadmill systems are known to induce both spatial and temporal constraints on gait and, as such, data may not be representative of unconstrained walking outside of the laboratory setting. Hence further research is needed to establish the accuracy and validity of the system for comparing different cohorts or establishing potential intervention effects. None–the–less, the findings of the current study provide clinicians and researchers with an indication of the sensitivity of the new Zebris treadmill system to detect changes in common spatiotemporal gait parameters and vertical ground reaction forces during walking and highlight the need for continued evaluation of the measurement characteristics of instrumented treadmill systems.

## Conclusions

The findings of present study demonstrate that small but statistically significant differences arise with repeated measurement of spatiotemporal and kinetic gait parameters in healthy young adults walking at self–selected ‘comfortable’ speeds when measured by an instrumented treadmill integrated with a pressure platform. The minimum change that can be detected with 95% confidence by the instrumented treadmill ranged between 3% and 17% for temporal parameters, 4% and 20% for kinetic parameters, and 14% and 33% for spatial parameters between days. Within–day repeatability was similar to that observed between–days. While temporal parameters were typically more consistent than spatial gait parameters, the findings highlight the need for continued evaluation of the measurement characteristics of the new instrumented treadmill system in a variety populations and over a wide range of gait speeds.

## Competing interests

The authors declare that they have no competing interests.

## Authors’ contributions

LFR conceived of the study, and participated in its design and coordination, assisted with data collection, and drafted the manuscript. SRU participated in the design of the study and carried out data collection. SCW performed statistical analysis and drafted the manuscript. All authors read and approved the final manuscript.

## Pre-publication history

The pre-publication history for this paper can be accessed here:

http://www.biomedcentral.com/1471-2474/14/249/prepub
